# Microbial Community Dynamics during the Bioremediation Process of Chlorimuron-Ethyl-Contaminated Soil by *Hansschlegelia* sp. Strain CHL1

**DOI:** 10.1371/journal.pone.0117943

**Published:** 2015-02-17

**Authors:** Liqiang Yang, Xinyu Li, Xu Li, Zhencheng Su, Chenggang Zhang, Huiwen Zhang

**Affiliations:** 1 State Key Laboratory of Forest and Soil Ecology, Institute of Applied Ecology, Chinese Academy of Sciences, Shenyang, China; 2 University of Chinese Academy of Sciences, Beijing, China; Netherlands Institute of Ecology (NIOO/KNAW), NETHERLANDS

## Abstract

Long-term and excessive application of chlorimuron-ethyl has led to a series of environmental problems. Strain *Hansschlegelia* sp. CHL1, a highly efficient chlorimuron-ethyl degrading bacterium isolated in our previous study, was employed in the current soil bioremediation study. The residues of chlorimuron-ethyl in soils were detected, and the changes of soil microbial communities were investigated by phospholipid fatty acid (PLFA) analysis. The results showed that strain CHL1 exhibited significant chlorimuron-ethyl degradation ability at wide range of concentrations between 10μg kg^-1^ and 1000μg kg^-1^. High concentrations of chlorimuron-ethyl significantly decreased the total concentration of PLFAs and the Shannon-Wiener indices and increased the stress level of microbes in soils. The inoculation with strain CHL1, however, reduced the inhibition on soil microbes caused by chlorimuron-ethyl. The results demonstrated that strain CHL1 is effective in the remediation of chlorimuron-ethyl-contaminated soil, and has the potential to remediate chlorimuron-ethyl contaminated soils in situ.

## Introduction

As a member of the sulfonylurea family, chlorimuron-ethyl is extensively used in soybean for inhibiting broadleaved weeds [1–3]. However, long-term and excessive application of this herbicide could raise series of environmental problems [4, 5]. On the one hand, it has led to soil degradation and crop rotation barriers. On the other hand, it has potential inhibitory effect on soil microbes, most of which play an important role in energy flow and nutrient cycle. To eliminate the chlorimuron-ethyl residue in the soil and water, the fate and behavior of this herbicide got increasingly attention. Its adsorption, desorption, toxicity and degradation in soil and water has been studied previously [6–8]. A series of microbes that can degrade this herbicide were isolated [9, 10]. However, most of these reports focused on degradation ability in culture condition. Few studies investigated the changes of soil microorganisms during the bioremediation process.

Hansschlegelia sp. CHL1, an efficient chlorimuron-ethyl degradation bacterium, was isolated in our previous study [11]. The aim of the present work was to assess the bioremediation ability of strain CHL1 by investigating the residues and microbial community dynimics during the remediation process. The total microbial biomass and community structure were studied by total PLFAs and indicator PLFA (GN/GP, bacteria/fungi PLFA, etc.) respectively. The findings of this study will contribute to optimization of bioremediation for chlorimuron-ethyl contaminated soils *in situ*.

## Materials and Methods

### Ethics statement

No specific permits were required for the described field studies since these locations are not privately-owned or protected in any way. Field studies did not involve endangered or protected species.

### Soil sampling

A surface soil (0–15cm) of abandoned land that had not been previously treated with herbicides and fertilizers for 16 years was collected at the National Field Research Station of Shenyang Agroecosystems, Shenyang city, China (41°47′N, 123°23′E). According to the FAO Soil Classification, soil was classified as aquic brown soil and its physical and chemical properties were as following: 13.7g kg^-1^ organic matter, 26.3 mg kg^-1^ available nitrogen (N), 10.6 mg kg^-1^ available phosphorus (P), and 133.7 mg kg^-1^ available potassium (K), with a pH of 6.52. After mixed and sieved through a 2-mm mesh, the soil was stored at room temperature until experimental treatments in the next day.

### Chemicals and media

Chlorimuron-ethyl and reagents used for chromatographic and spectroscopic analysis were all purchased from Sigma-Aldrich Chemical Co. (Shanghai, China). All other chemicals and solvents were of analytical grade. Strain *Hansschlegelia* sp. CHL1 was isolated in our previous research.

Phosphate-basal minimal medium (PBM) contained 0.5 g NaNO_3_, 1.0 g (NH_4_)_2_SO_4_, 2.5 g Na_2_HPO_4_, 1.0 g KH_2_PO_4_, and 1 mL of mineral solution per liter [12]. PBMM medium consisted of PBM supplemented with methanol (10 mL L^-1^).

### Experimental design and treatments

The soil was split into twelve groups and treated separately as described in Table 1. For each treatment, 300g of soils was put in a pot (diameter, 10cm; depth, 15cm) with 3 replications. Strain CHL1 was cultured in PBMM liquid medium till stationary phase and then harvested by centrifugation at 4°C (10000× *g*, 10 min). After removing the supernatant, the cell pellets were washed twice and suspended (OD_600_ 3.0) with PBS (0.2 mol L^-1^, pH 7.8). The final concentration of strain CHL1 in soil was 1.1×10^8^ CFU g^-1^. Sterile deionized water was added to adjust the soil moisture to 20%.

**Table 1:** The list of all treatments.

Treatment Code	Chlorimuron-ethyl concentration (μg kg^-1^)	Strain CHL1
S_0-_	0	uninoculated
S_0+_	0	inoculated
S_10-_	10	uninoculated
S_10+_	10	inoculated
S_30-_	30	uninoculated
S_30+_	30	inoculated
S_50-_	50	uninoculated
S_50+_	50	inoculated
S_100-_	100	uninoculated
S_100+_	100	inoculated
S_1000-_	1000	uninoculated
S_1000+_	1000	inoculated

The pots were incubated at 25°C for 2 months in a dark room. Throughout the incubation period, sterile deionized water was added to maintain the soil moisture at 20% (±5%). Soil samples were periodically removed for chlorimuron-ethyl residue determination and PLFA analysis on days 1, 7, 15, 30, 45 and 60.

### Determination of residual chlorimuron-ethyl in soils

The residual chlorimuron-ethyl in soils were determinated as previous report [13]. Briefly, a 10g soil sample was weighed into a 50mL polystyrene tube and extracted with 10mL mixture solution of PBS (pH 7.8) and acetonitrile (8:2, v/v). After shaken at 150rpm for 20min on a rotary shaker, the mixture was centrifuged (4000×g, 5min). The extraction was repeated thrice and the supernatants were merged and acidified to pH 2.5. A Cleanert HXN cartridge (500mg 6mL^-1^, Agela Technologies Inc.) was used to purify the residue. The elution was dried under N_2_, resuspended in 1mL methanol and filtered through a 0.22μm nylon filter. The residual of chlorimuron-ethyl in different treatment soils were analyzed by HPLC equipped with a Zorbax C-18 ODS Spherex column (4.6 × 250 mm, 5 μm, Agilent Technologies, Palo Alto, CA, USA). Detection of chlorimuron-ethyl was performed at 254nm with a mobile phase consisting of 0.5% acetic acid: methanol (30:70, v/v) at a flow rate of 1mL min^-1^ [14, 15]. 10μL of each solution was injected into the HPLC system for detection.

### Analysis of soil microbial community structure

Phospholipid fatty acid (PLFA) analysis was employed to determine the soil microbial community structure. Soil lipids extraction was carried out as described by Petersen and Klug [16], with minor modifications. Briefly, 8g freeze-dried soil sample was added in a Teflon screw cap culture tube and extracted with 30.4 mL mixture of MeOH/CHCl_3_/citric acid buffer (0.15M, pH 4) (2:1:0.8,v/v/v). The extraction was implemented twice and the CHCl_3_ layer was then collected and dried under N_2_.

A Cleanert Silica cartridge (500mg 6mL^-1^, Agela Technologies Inc.) was employed to fractionate polar lipids from glycol- and neutral. The polar lipids were transformed into fatty acid methyl esters (FAMEs) by methylation. The individual FAME in different treatment soils were analyzed by an Agilent 7890GC equipped with a HP-5 MS column (60m × 0.25 mm inner diameter, 0.25μm, Agilent Technologies, Palo Alto, CA, USA). The results were analyzed by using the MIDI Sherlock system (MIDI, Newark, DE, USA). An internal standard 19:0 peak was used to quantify fatty acids by comparison of the peak areas.

Fatty acids with carbon chains ranging from 12 to 20 carbon atoms are generally associated with microorganisms and were grouped into Bacteria, Fungi, Actinobacteria, Gram-positive (GP) and Gram-negative (GN) as following. Fatty acids used as markers for bacteria were i15:0, 15:0, a15:0, i16:0, 16:1ω7, i17:0, a17:0, cy17:0, 17:0 and cy19:0 [17, 18]. Fatty acids 18:2ω6 and 18:1ω9c were used as fungal markers [19, 20] while 10Me17:0, 10Me18:0 [21]were used as indicator of actinobacteria. The branched phospholipids i14:0, i15:0, a15:0, i16:0, i17:0 and a17:0 were used as GP bacteria marker while the GN bacteria biomass were assessed by cy17:0, cy19:0, 17:1ω9c, 16:1ω7c, and 18:1ω9c [18, 22]. The total amount of PLFA was calculated to indicate the total microbial biomass. The rates of bacteria/fungal (B/F) and GN/GP were calculated to indicate the relative changes of major microbial groups [23]. The stress level of microbial community was assessed by the Stress index, calculated from the ratio of (cyc17:0+cyc19:0)/ (16:1ω7c+18:1ω7c) [24].

### Data analysis and statistics

All values reported in this paper are the mean (±SE) of three replicates. Two-way and three-way ANOVA were carried out using SPSS 16.0 (SPSS, Chicago, lllinois, USA) to compare the significant effects (p<0.05*, p<0.01**, or p<0.001***) and mean separations were conducted using Tukey′s test. The PLFA data were log transformed to meet the assumptions of normality and homoscedasticity (Kolmogorov-Smirnov and Levene tests, respectively). Principal components analysis (PCA) was performed using Canoco4.5 (Microcomputer Power, Ithaca, NY, USA).

## Results

### Chlorimuron-ethyl degradation in experimental soils

The residues of chlorimuron-ethyl in experimental soils with or without the addition of strain CHL1 were determinated (Table 2). The degradation dynamics were analyzed and the results indicated that the degradation of chlorimuron-ethyl in soil was accordance with the first-order kinetic equation C = C_0_e^-kt^, where C is the concentration of chlorimuron-ethyl, t is the time interval, e is the Euler’s constant and C_0_ is the constant. However, there was a significant difference in the half-life of chlorimuron-ethyl between soils inoculated with strain CHL1 or not. For the treatments without strain CHL1, the chlorimuron-ethyl residue was under the minimum detectable level in the 10μg kg^-1^ treated soil after 45 days. Higher initial concentration of chlorimuron-ethyl had much longer half-life. At the end of the incubation period, the chlorimuron-ethyl residues were 4.43μg kg^-1^, 11.42μg kg^-1^, 44.3μg kg^-1^ and 524.36μg kg^-1^ in the 30μg kg^-1^, 50μg kg^-1^, 100μg kg^-1^ and 1000μg kg^-1^ chlorimuron-ethyl-treated soils, respectively. The rate of chlorimuron-ethyl degradation increased significantly when strain CHL1 was inoculated in the soils. The chlorimuron-ethyl residues were under the minimum detectable level in the 10μg kg^-1^, 30μg kg^-1^ and 50μg kg^-1^ treatment soils with strain CHL1 inoculation after 15, 30 and 45 days respectively. In addition, the inoculated soils with high chlorimuron-ethyl concentration (100μg kg^-1^ and 1000μg kg^-1^) showed significant lower chlorimuron-ethyl residues than the non-inoculated soils.

**Table 2:** The degradation of chlorimuron-ethyl in treated soils.

Tested soils	1d	7d	15d	30d	45d	60d	Degradationcurve	Half-life
S_10-_ *	9.94±0.81	8.35±0.89	7.12±0.71	4.31±1.08	0	0	C = 10.369e^-0.029x^	25.17d
S_30-_	29.75±1.25	23.71±1.49	18.36±1.23	10.51±1.18	6.02±0.64	4.43±1.16	C = 29.798e^-0.033x^	20.80d
S_50-_	49.91±1.25	41.65±1.72	35.29±1.17	25.47±1.26	18.38±1.05	11.42±1.32	C = 50.899e^-0.024x^	29.62d
S_100-_	100.05±3.21	89.26±3.17	76.18±2.44	62.62±2.51	51.68±1.86	44.3±1.57	C = 97.343e^-0.014x^	47.57d
S_1000-_	1002.75±22.08	923.47±19.34	869.25±19.07	750.35±17.76	639.072±11.06	524.36±14.16	C = 1014.1e^-0.011x^	64.27d
S_10+_	9.95±1.12	3.51±0.63	0	0	0	0	C = 7.5613e^-0.077x^	5.37d
S_30+_	29.81±1.02	11.43±3.15	5.07±0.41	0	0	0	C = 31.33e^-0.125x^	5.89d
S_50+_	49.87±1.31	26.98±2.25	15.47±2.18	7.61±1.51	0	0	C = 45.86e^-0.063x^	9.63d
S_100+_	98.71±3.67	83.85±5.51	61.96±6.82	31.19±4.06	17.22±4.32	10.26±3.2	C = 106.8e^-0.04x^	18.97d
S_1000+_	999.47±22.58	725.92±31.49	526.75±46.15	418.94±37.02	307.44±44.92	220.7±39.13	C = 878.9e^-0.024x^	23.50d

Values represent the mean (±SD) of three replicates.

*S_10_, S_30_, S_50_, S_100_, S_1000_ mean 10, 30, 50, 100, 1000μg kg^-1^ chlorimuron-ethyl treatment group, respectively. ‘-’ indicates without strain CHL1 inoculation; ‘+’ indicates inoculation with strain CHL1.

### Statistical analysis of PLFA concentration

A three-way ANOVA for total PLFAs, bacteria/fungi ratios, GN/GP ratios, stress level and the Shannon index was employed to identify the significant effect of inoculation, time and treatment. The results showed that single factors and their interactions all had significant effect on total PLFAs, bacteria/fungi ratios, GN/GP ratios, stress level and the Shannon index (Table 3).

**Table 3:** Multivariate analysis of variance by three-way ANOVA of the sum of all PLFAs, ratios of GN/GP and bacteria/fungi, the stress level and the Shannon index.

Facter*	Total PLFA	GN/GP	Bacteria/fungi	Stress	Shannon index
Inoculation(In)	364.483***	197.060***	111.661***	699.121***	42.567***
Treatment	382.084***	390.110***	33.038***	372.016***	106.028***
Time	231.672***	70.420***	221.238***	36.333***	32.437***
In×treat	57.163***	96.359***	12.873***	67.431***	18.360***
In×time	21.972***	11.654***	24.394***	29.171***	4.647**
Treat×time	18.833***	27.332***	26.963***	19.524***	75.766***
In×treat×time	7.780***	22.048***	17.482***	12.710***	7.202***

*The categorical factors are inoculation with strain CHL1, treatment (S_0_, S_10_, S_30_, S_50_, S_100_, S_1000_), incubation time (1, 7, 15, 30, 45, 60 days). Presented are the F-values with the level of significance.

### The changes of total PLFA in experimental soils

**Fig 1:**
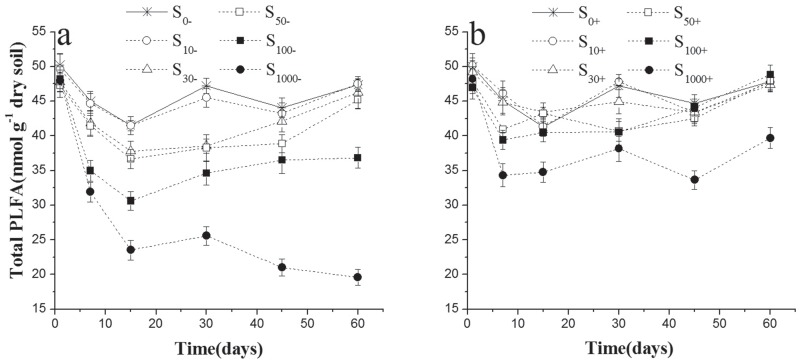
Values of total PLFAs biomass in treated soils inoculation with strain CHL1 (b) or not (a). Symbols represent the mean of triplicate samples and error bars indicate the standard deviation. S_10_, S_30_, S_50_, S_100_, S_1000_ mean 10, 30, 50, 100, 1000μg kg^-1^ chlorimuron-ethyl treatment group, respectively. ‘-’ indicates without strain CHL1 inoculation; ‘+’ indicates inoculation with strain CHL1.

The total PLFA concentrations of soils without inoculation ranged from 21.02 to 50.21 nmol g^-1^ over the incubation period and were significantly (F_inoculation_ = 364.48, p<0.001) lower than that of inoculated soils (33.62–50.19 nmol g^-1^) (Fig. 1, Table 3). Chlorimuron-ethyl showed persistent inhibitory effect on the total PLFAs and this effect was dose-dependent (Fig. 1a): 10μg kg^-1^ chlorimuron-ethyl (S_10-_) had no significant effect on the total PLFAs compared with uncontaminated soil (S_0-_), and the total PLFAs in the 30μg kg^-1^and 50μg kg^-1^ chlorimuron-ethyl soils (S_30-_ and S_50-_) showed an obvious decline over the first two weeks and were capable of self-restoration by the end of the incubation (Fig. 1a). However, the total PLFAs were significantly decreased in soils contaminated with 100μg kg^-1^ and 1000μg kg^-1^ chlorimuron-ethyl throughout the experiment (S_100-_ and S_1000-_) (Fig. 1a). Once inoculated into the soils, strain CHL1 could significantly relieve the negative effect of chlorimuron-ethyl both in degree and duration (Fig. 1b): The total PLFAs in inoculated soil treated with less than or equal to 100μg kg^-1^ of chlorimuron-ethyl had recovered to the control level since day 45. Even in the 1000μg kg^-1^ treatment, total PLFAs in inoculated soils increased significantly than non-inoculated soils (S_100-_) since day 15, though total PLFAs were not able to recover to the control level at the end of the incubation.

### The changes of GN/GP bacterial PLFA ratio in experimental soils

**Fig 2:**
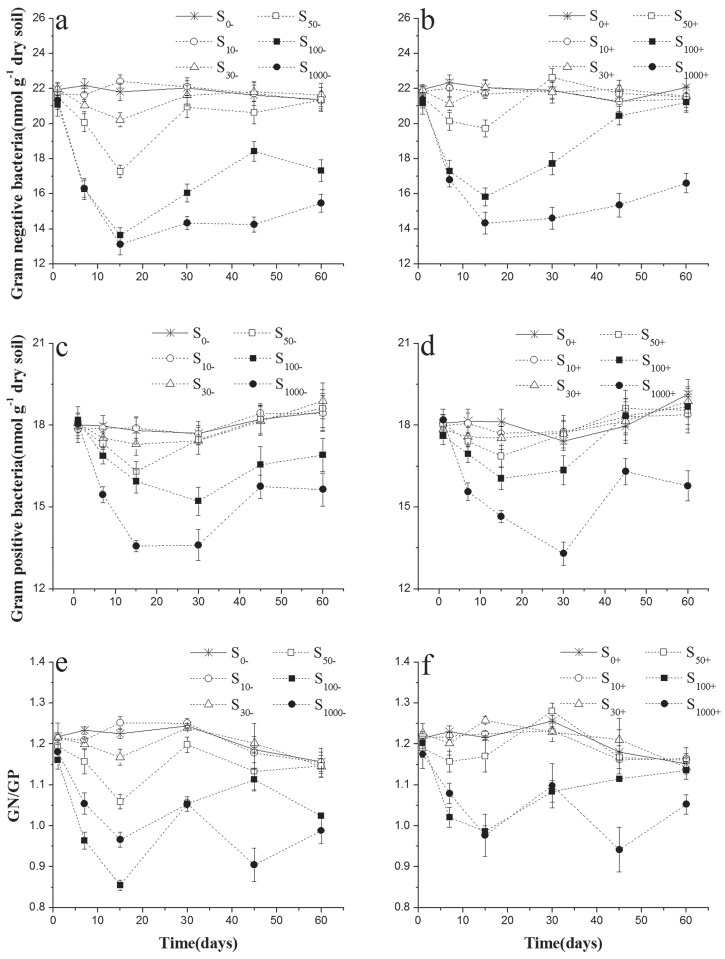
Values of Gram negative bacteria (GN), Gram positive bacteria (GP) and the rate of GN and GP in treated soils inoculation with strain CHL1 (b, d, f) or not (a, c, e). Symbols represent the mean of triplicate samples and error bars indicate the standard deviation. S_10_, S_30_, S_50_, S_100_, S_1000_ mean 10, 30, 50, 100, 1000μg kg^-1^ chlorimuron-ethyl treatment group, respectively. ‘-’ indicates without strain CHL1 inoculation; ‘+’ indicates inoculation with strain CHL1.

Compared with the uncontaminated soil, both PLFA concentrations of GN and GP bacteria markers in chlorimuron-ethyl-treated soils decreased in the early stage of incubation, and GN bacteria were inhibited by chlorimuron-ethyl more severely than GP bacteria (Fig. 2a, c). The decrease effects caused by chlorimuron-ethyl contamination were dose-dependent. At the end of incubation, the PLFA concentrations of GN and GP bacteria markers gradually recover to the control level in the 10–50μg kg^-1^chlorimuron-ethyl-treated soils without inoculation (Fig. 2a, c). Once inoculated into the soils, strain CHL1 could significantly relieve the negative effect of chlorimuron-ethyl for both GN and GP bacteria, and the PLFA concentrations of GN and GP bacteria markers gradually return to the control level in the 10–100μg kg^-1^chlorimuron-ethyl-treated soils at the end of incubation (Fig. 2b, d). So the ratio of GN/GP PLFA markers of chlorimuron-ethyl-treated soils tended to decrease significantly at the early of stage, and the inhibited effects were dose-dependent (F_treatment_ = 390.11, p<0.01) (Fig. 2e, Table 3). The inoculation of strain CHL1 relieved significantly the inhibited effect of chlorimuron-ethyl on GN/GP ratio ((F_inoculation_ = 197.060, p<0.001) (Fig. 2f, Table 3). In soils without strain CHL1 inoculation, the ratio of GN/GP in chlorimuron-ethyl-treated soils could recover to the control level at the end of incubation except for 100 and 1000μg kg^-1^ treatment (Fig. 2e). When there was strain CHL1 inoculation, the ratio of GN/GP could recover to the control level except for 1000μg kg^-1^ treatment (Fig. 2f).

### The changes of bacteria/fungi PLFA ratio in experimental soils

**Fig 3:**
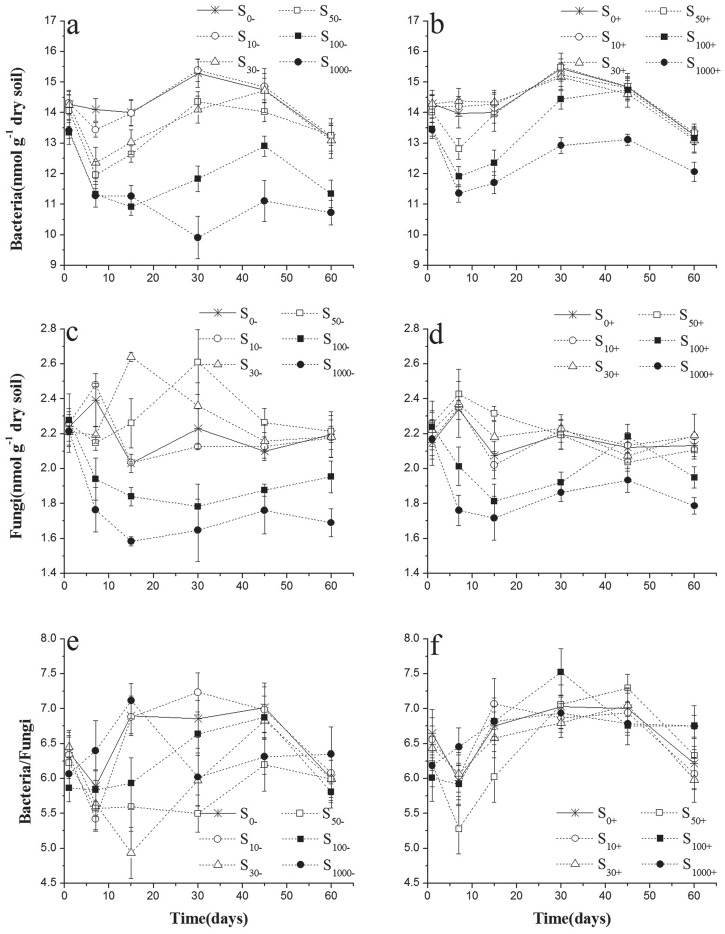
Values of bacteria, fungi and the rate of bacteria and fungi in treated soils inoculation with strain CHL1 (b, d, f) or not (a, c, e). Symbols represent the mean of triplicate samples and error bars indicate the standard deviation. S_10_, S_30_, S_50_, S_100_, S_1000_ mean 10, 30, 50, 100, 1000μg kg^-1^ chlorimuron-ethyl treatment group, respectively. ‘-’ indicates without strain CHL1 inoculation; ‘+’ indicates inoculation with strain CHL1.

Chlorimuron-ethyl showed persistent inhibition to bacteria in a dose dependent manner (Fig. 3a). However, on fungi, low dose of chlorimuron-ethyl exhibited positive effect and high concentration of chlorimuron-ethyl showed negative effect (Fig. 3c). The ratio of bacteria and fungi, therefore, decreased at first and then got up with the increasing concentration of chlorimuron-ethyl (F_treatment_ = 33.038, p<0.001) (Fig. 3e, Table 3). This negative effects on bacteria fungi, and ratios of bacteria and fungi could be significantly relieved with the inoculation of strain CHL1 (F_inoculation_ = 111.661, p<0.001) (Fig. 3b, d, f). Although the PLFA concentrations of bacteria and fungi markers in 10–50μg kg^-1^chlorimuron-ethyl-treated soils without inoculation could be self-restoration by the end of the incubation (Fig. 3a, c), inoculation reduced the time to recover, and there was no obvious difference between the treatments and the control since day 15 (Fig. 3b, d). By inoculation, the PLFA concentrations of bacteria and fungi markers in 100μg kg^-1^ chlorimuron-ethyl-treated soils recovered to the control level at the end of incubation (Fig. 3b, d). So, the ratios of bacteria and fungi in soils treated with strain CHL1 were more stable in all the concentration of chlorimuron-ethyl throughout the experiment (Fig. 3f), which was significantly important to the circulation of materials and energy metabolism in soil.

### The changes of stress indices in experimental soils

**Fig 4:**
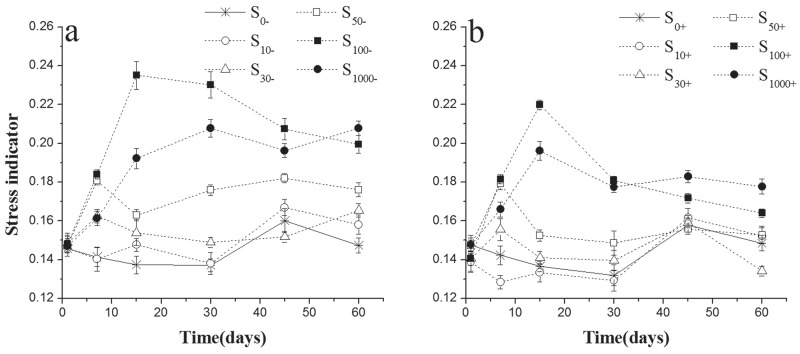
Values of the stress level of microbes in treated soils inoculation with strain CHL1 (b) or not (a). Symbols represent the mean of triplicate samples and error bars indicate the standard deviation. S_10_, S_30_, S_50_, S_100_, S_1000_ mean 10, 30, 50, 100, 1000μg kg^-1^ chlorimuron-ethyl treatment group, respectively. ‘-’ indicates without strain CHL1 inoculation; ‘+’ indicates inoculation with strain CHL1.

In our study, the addition of chlorimuron-ethyl significantly increased the stress level of microbes in soils and this effect was dose-dependent excepted for 1000μg kg^-1^ treatment groups (F_treatment_ = 372.02, p<0.001) (Fig. 4, Table 3). Incubation time had significant effect on the stress level of microbes in all treatments soils (F_time_ = 36.33, p<0.001) (Fig. 4, Table 3). The stress level of microbes in soils treated with 10–50μg kg^-1^ chlorimuron-ethyl tended to be higher than in the control samples at the first 30days. And this change could be self-restoration by the end of the incubation period in soils with or without the addition of strain CHL1 (Fig. 4). However, the stress level became much higher than control level in high concentration (100μg kg^-1^ and 1000μg kg^-1^) of chlorimuron-ethyl treatment soils and could not self-recover by the end of experiment period. There was a significant decline (F_inoculation_ = 699.12, p<0.001) (Fig. 4, Table 3) in the stress level of microbes among treatments with regards to the addition of strain CHL1.

### The changes of Shannon-Wiener indices in experimental soils

**Fig 5:**
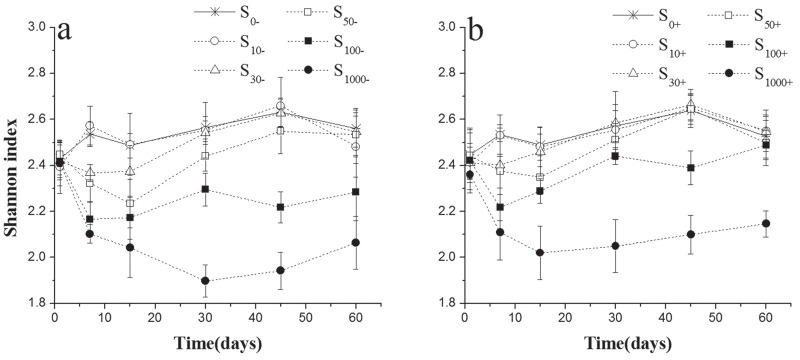
Values of the Shannon-Wiener index of microbes in treated soils inoculation with strain CHL1 (b) or not (a). Symbols represent the mean of triplicate samples and error bars indicate the standard deviation. S_10_, S_30_, S_50_, S_100_, S_1000_ mean 10, 30, 50, 100, 1000μg kg^-1^ chlorimuron-ethyl treatment group, respectively. ‘-’ indicates without strain CHL1 inoculation; ‘+’ indicates inoculation with strain CHL1.

The Shannon-Wiener indices of microbes in soils with or without the addition of strain CHL1 were investigated (Fig. 5). Both the treatments (F_treatment_ = 106.03, p<0.001) (Fig. 5, Table 3) and incubation time (F_time_ = 326.44, p<0.001) had significant effects on the Shannon-Wiener index, regardless of inoculation with strain CHL1 or not. The indices ranged from 1.90 to 2.66 for treatment soils without the strain CHL1 inoculation during the whole incubation (Fig. 5a). Chlorimuron-ethyl decreased the soil microbial diversity in dose-dependent manner. This decrease could be self-recovery in low and median dose (15μg kg^-1^, 30μg kg^-1^ and 50μg kg^-1^) of chlorimuron-ethyl treatment by the end of experiment period. However, high dose (100μg kg^-1^ and 1000μg kg^-1^) of chlorimuron-ethyl showed more severe and persistent negative effect on the Shannon-Wiener indices. In soils with strain CHL1 inoculation (Fig. 5b), the Shannon-Wiener indices of microbes were significantly higher than non-inoculation treatment (F_inoculation_ = 42.57, p<0.001) (Table 3).

### Microbial community structures in experimental soils

**Fig 6:**
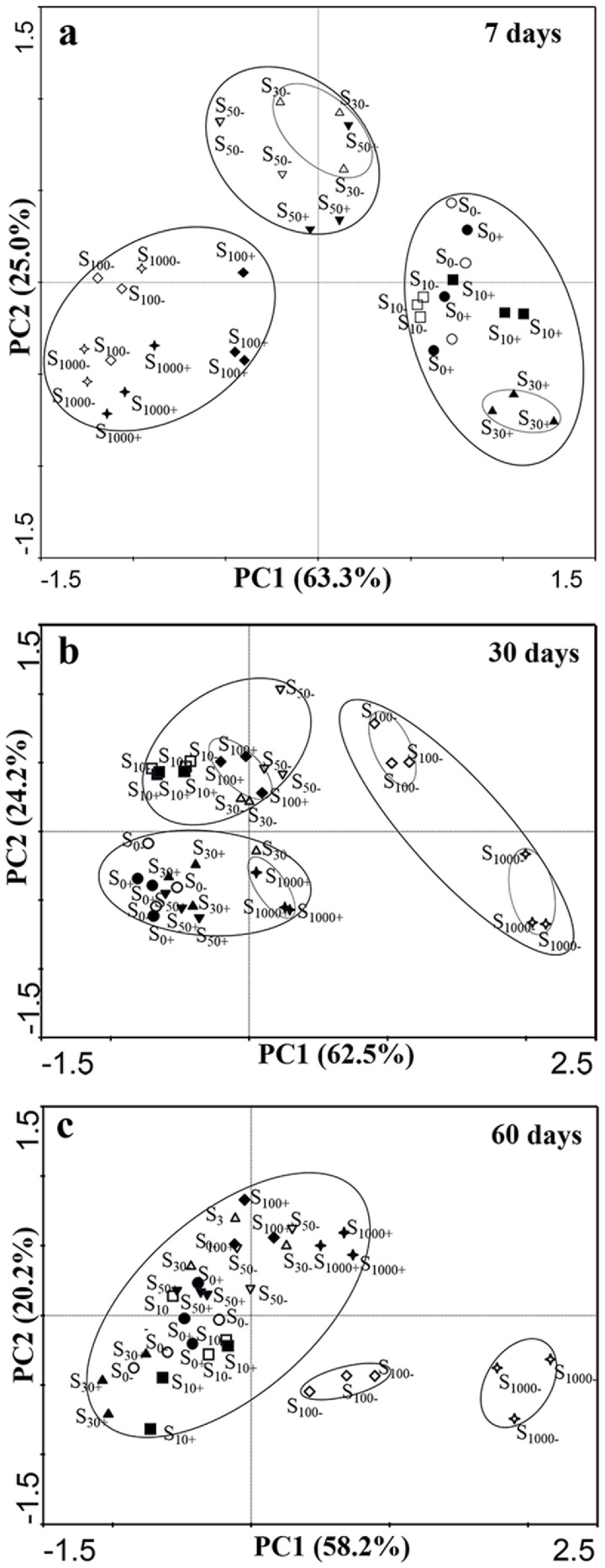
PCA plot of the microbial community structure of all treatments on days 7, 30 and 60. S_0_, S_10_, S_30_, S_50_, S_100_ and S_1000_ mean 0, 10, 30, 50, 100 and 1000μg kg^-1^ chlorimuron-ethyl treatment group, respectively. ‘-’ indicates without strain CHL1 inoculation; ‘+’ indicates inoculation with strain CHL1.

The principal component analysis (PCA) of the PLFA patterns employed 18 most common PLFAs and the results were presented in a series of three plots in Fig. 6. S_0_, S_10_, S_30_, S_50_, S_100_ and S_1000_ mean 0, 10, 30, 50, 100 and 1000μg kg^-1^ chlorimuron-ethyl treatment group, respectively. ‘-’ indicates without strain CHL1 inoculation; ‘+’ indicates inoculation with strain CHL1. The differences of PLFA patterns in all treatment soils on the 7^th^ day were showed in Fig. 6a. Twelve treatments were split into three groups which contained samples with low (S_0_, S_10_ and S_30+_) medium (S_30-_, S_50_) and high (S_100_, S_1000_) chlorimuron-ethyl concentrations treatments, respectively. The result indicated that chlorimuron-ethyl had significant effect on PLFA patterns in a dose-dependent way. However, the treatment groups (S_30_) in 30μg kg^-1^ chlorimuron-ethyl concentration were of exception. Treatment S_30-_ represented 30μg kg^-1^ chlorimuron-ethyl treated soils without inoculation, and was clustered into a group with the samples of 50μg kg^-1^ chlorimuron-ethyl treatment (S_50_), but the S_30+_ treatment represented 30μg kg^-1^ chlorimuron-ethyl treated soils with strain CHL1 inoculation, which was found in the low-dose group including S_0_ and S_10_ chlorimuron-ethyl-treated samples. The same condition happened in 100 and 1000μg kg^-1^ chlorimuron-ethyl-treated samples with inoculation (S_100+_, S_1000+_) or not (S_100-_ and S_1000-_) on the 30^th^ day (Fig. 6b), which illustrated that strain CHL1 has significant effect on relieving the inhabitation on PLFA patterns caused by chlorimuron-ethyl. On the 60^th^ day, the effect of chlorimuron-ethyl reduced with the incubation time and inoculation application, and most of the treatment were gathered together except for treatment S_100-_ and S_1000-_, which represented 100μg kg^-1^ and 1000μg kg^-1^ chlorimuron-ethyl-treated samples without inoculation, respectively (Fig. 6c).

## Discussion

### Chlorimuron-ethyl degradation in experimental soils

Chemical hydrolysis and microbial degradation were considered to be the major degradation process for sulfonylureas [25, 26]. However, this process is particularly dependent on soil temperature, moisture, organic matter and pH [6]. Bioaugmentation has been proven to be an efficient technique for soil bioremediation [27, 28]. A series of microbes showed high chlorimuron-ethyl degradation ability in culture condition. However, most of these strains used chlorimuron-ethyl as a nitrogen source but not sole carbon and energy source. Zhang et al. [15] indicated that strain LF1 could grow with chlorimuron-ethyl as nitrogen source and degraded 77% of the 5 mg L^-1^ chlorimuron-ethyl after incubation for 4 days. Ma et al. [14] described that when chlorimuron-ethyl was provided as the sole nitrogen source, the degradation efficiency in liquid medium was about 81.0% after 7 days of inoculation with strain LW3; when used as the sole carbon source, only 25% of the initially added chlorimuron-ethyl was degraded in the same time span. Former research showed that when chlorimuron-ethyl was provided as the sole carbon source, more than 95% of chlorimuron-ethyl at an initial concentration of 50 mg L^-1^ was degraded by strain CHL1 at the end of a 2-day incubation period at 30°C [11]. Compared with medium condition, strongly influenced by competition of indigenous microorganisms and the suitability of the environment, the survival of degradation microorganisms and their chlorimuron-ethyl degradation efficiencies were severely inhibited in soil. Few strains showed high chlorimuron-ethyl degradation ability in soil condition. By determing the chlorimuron-ethyl residue and investigating the survival time of degrading strain CHL1 in soils, we concluded that strain CHL1 maintained a high copy number and showed high degradation efficiency in 5mg kg^-1^ chlorimuron-ethyl contaminated soil in former research [11]. In this experiment, strain CHL1 displayed high chlorimuron-ethyl degradation ability, both in low and high degree of chlorimuron-ethyl contamination soils. The results demonstrated that strain CHL1 could be promising for chlorimuron-ethyl contaminated soil remediation in situ.

### PLFA concentration

Due to their rapid turnover in soils, the total PLFAs is a good indicator of the active microbial biomass [29, 30]. Previous researches have proven that the residuals of sulfonylurea herbicides in cropland have negative effects on the microbes in a dose-dependent way [6, 31]. Owing to the chemical hydrolysis and degradation by indigenous microorganism, the chlorimuron-ethyl contaminated soils without inoculation of strain CHL1 could self-recovery within a certain range [7]. However, this process was agonizingly slow and limited. Some microbes sensitive to chlorimuron-ethyl might be seriously inhibited at this period. In this study, strain CHL1 exhibited high efficient and persistent remediation ability in different concentration of chlorimuron-ethyl contaminated soils. Throughout the incubation period, the total PLFA concentrations in soils with inoculation of strain CHL1 were more stable and analogous to the control level, which was of significantly important for maintaining the sustainability and productivity of the soil ecosystem [32].

As the different structure of peptidoglycan, Gram negative bacteria are more sensitive than Gram positive bacteria with environmental variation [33]. Former researches have showed that low dose of herbicides or pesticides significantly increased soil GN bacteria; but at high concentrations, the abundance of GN would decline [34, 35]. Our study exhibited the same trend. In low and median dose (30μg kg^-1^, 50μg kg^-1^, 100μg kg^-1^) of chlorimuron-ethyl treatment soils, GN bacteria were more severely inhibited and the ratio of GN: GP decreased. With the increase of chlorimuron-ethyl concentrations (1000μg kg^-1^), both GN and GP were seriously inhibited and the ratio of GN: GP got up. Compared with non-inoculation treatments, the ratios of GN: GP in soils inoculated with strain CHL1 were more stable, even the concentration of chlorimuron-ethyl being as high as 100μg kg^-1^. The ratio of GN: GP in 1000μg kg^-1^ chlorimuron-ethyl treatment did not recover to the control level regardless of CHL1 inoculation, which was corresponding to the result of chlorimuron-ethyl degradation in experimental soils.

Bacteria and fungi usually showed differently sensitive to herbicides. He et al. demonstrated that the aerobic heterotrophic bacteria was distinctly inhibited and the number of tolerant fungi increased greatly in the rhizosphere after the application of metsulfuron-methyl [36]. Said et al. showed that long-term application of urea herbicides clearly affected both the structure and metabolic of the soil microbial communities [37]. Furthermore, some beneficial indigenous microbes might be replaced by pathogenic bacterial which are more resistant to excess herbicide [5, 38].

Former researches have illustrated that the cis-monoenoic fatty acids will be preferentially utilized in severe environment [39, 40]. To detect shifts in microbial community due to the toxicity of chlorimuron-ethyl, the ratio of (cyc17:0+cyc19:0)/ (16:1ω7c+18:1ω7c) was used as the indicator of microbial community stress level in soils. In our study, chlorimuron-ethyl significantly increased the stress index, especially in high concentration. However, the stress index decreased obviously by the addition of strain CHL1, in all chlorimuron-ethyl treatments. The stress indices in 30μg kg^-1^ and 50μg kg^-1^ treatment soils with strain CHL1 inoculation slightly rose at the first month and recovered to the control level soon. 100μg kg^-1^ of chlorimuron-ethyl treatment showed the highest stress index in most incubation period, regardless of inoculation with strain CHL1or not. However, even in high concentration (100μg kg^-1^ and1000μg kg^-1^) of chlorimuron-ethyl treatments, strain CHL1 exhibited significant remediation ability.

Shannon-Wiener index is usually used to evaluate the evenness of species representation [41, 42]. Chlorimuron-ethyl decreased the diversity and evenness of soil microbial community by long-term utilization [5, 43]. Our results were in accordance with these reports. Chlorimuron-ethyl decreased the soil microbial diversity in dose-dependent manner. Though the indigenous microbes showed self-recovery ability at low chlorimuron-ethyl concentration, this process was agonizingly slow and limited especially in high chlorimuron-ethyl degree. In soils with strain CHL1 inoculation (Fig. 5b), this inhibition effect caused by chlorimuron-ethyl was obviously relieved, both in degree and duration. Even in 100μg kg^-1^ chlorimuron-ethyl contaminated soil, the Shannon-Wiener diversity index recovered to a level equivalent to the control after 60 days incubation.

The principal component analysis (PCA) of the PLFA revealed significant differences in the PLFA pattern among the different treatments. Former researches have reported that herbicide treatments significantly influenced the PCA results [23, 44, 45]. In our study, the highest differences in microbial community among all treatments were observed on day 7 (Fig. 6a). On the 7^th^ day, nine fatty acids [16:1ω7, i17:0, a17:0, cy17:0, 17:0, cy19:0, 17:1ω9c (indicators of bacteria) and 16:1ω7c, 18:1ω9c (indicators of GN bacteria)] were strongly correlated (|r|≥0.6, |r| is absolute value of correlation coefficient [34].) to PC1, and another three fatty acids [18:2ω6, 18:1ω9c (indicators of fungi) and 18:1ω9t] were strongly correlated to PC2. Inoculation with strain CHL1 showed significant effects on PLFA patterns throughout the experiment period. On the 60^th^ day, the influences caused by chlorimuron-ethyl on the microbial communities were much little compared with day 7^th^, except for 100 and 1000μg kg^-1^ chlorimuron-ethyl and non-inoculation treatments.

## Conclusions

Strain CHL1, which was isolated from chlorimuron-ethyl-contaminated soil in our previous research, exhibited the significant chlorimuron-ethyl degradation ability at wide range of substrate concentration between 10μg kg^-1^ and 1000μg kg^-1^. The chlorimuron-ethyl concentrations were under the minimum detectable level in the 10μg kg^-1^, 30μg kg^-1^ and 50μg kg^-1^ treatments with strain CHL1 inoculation, after 15, 30 and 45days incubation, respectively. Even in the high chlorimuron-ethyl concentration treatments (100μg kg^-1^ and 1000μg kg^-1^), the inoculated soils showed a significant lower chlorimuron-ethyl residues than the non-inoculated soils. The effects of chlorimuron-ethyl on soil microbial communities were in a dose-dependant manner. High concentrations of chlorimuron-ethyl significantly decreased the total concentration of PLFA and the Shannon-Wiener indices and increased the stress level of microbes in soils. The inoculation with strain CHL1, however, reduced the inhibition on soil microbes caused by chlorimuron-ethyl. The biomarkers in soils treated with low and median dose of chlorimuron-ethyl could recover to the control level more quickly by inoculation than non-inoculation treatments. The levels of biomarkers did not recover to the level of control in high dose chlorimuron-ethyl treatments but were significantly relieved compared with non-inoculation treatments. These results showed the potential of strain CHL1 to remediate chlorimuron-ethyl contaminated soils in situ.
